# Global mental health: transformative capacity building in Nicaragua

**DOI:** 10.3402/gha.v6i0.21328

**Published:** 2013-09-30

**Authors:** Jaime C. Sapag, Andrés Herrera, Ruth Trainor, Trinidad Caldera, Akwatu Khenti

**Affiliations:** 1 Office of Transformative Global Health, Centre for Addiction and Mental Health, Toronto, Ontario, Canada; 2 Department of Psychiatry, University of Toronto, Toronto, Ontario, Canada; 3 Department of Family Medicine, Pontificia Universidad Católica de Chile, Santiago, Chile; 4 Centre for Demographic and Health Research (CIDS), National Autonomous University of Nicaragua in León (UNAN-León), León, Nicaragua; 5 Dalla Lana School of Public Health, University of Toronto, Toronto, Ontario, Canada

**Keywords:** Global mental health, primary healthcare, capacity building, Nicaragua, Latin America

## Abstract

**Background:**

Mental health is increasingly recognised as integral to good public health, but this area continues to lack sufficient planning, resources, and global strategy. It is a pressing concern in Latin America, where social determinants of health aggravate existing inequities in access to health services. Nicaragua faces serious mental health needs and challenges. One key strategy for addressing gaps in mental health services is building capacity at the primary healthcare and system levels.

**Objective:**

Using the framework of best practice literature, this article analyses the four-year collaborative process between the National Autonomous University of Nicaragua in León (UNAN-León) and the Centre for Addiction and Mental Health (CAMH) in Canada, which is aimed at improving mental healthcare in Nicaragua.

**Design:**

Based on a critical analysis of evaluation reports, key documents, and discussion among partners, the central steps of the collaboration are analysed and main successes and challenges identified.

**Results:**

A participatory needs assessment identified local strengths and weaknesses, expected outcomes regarding competencies, and possible methodologies and recommendations for the development of a comprehensive capacity-building programme. The partners delivered two international workshops on mental health and addiction with an emphasis on primary healthcare. More recently, an innovative Diploma and Master programme was launched to foster interprofessional leadership and effective action to address mental health and addiction needs. Collaborative activities have taken place in Nicaragua and Canada.

**Discussion:**

To date, international collaboration between Nicaragua and CAMH has been successful in achieving the jointly defined goals. The process has led to mutual knowledge sharing, strong networking, and extensive educational opportunities. Evidence of effective and respectful global health capacity building is provided. Lessons learned and implications for global health action are identified and discussed.

Mental health is increasingly recognised as integral to good public health (1, 2), but this area continues to lack sufficient planning, resources, and global strategy (3). The issue is particularly problematic in low- and middle-income countries (LMICs), where an estimated four out of five people with a serious mental illness do not receive appropriate treatment (4). It is a pressing concern in Latin America, where social determinants of health aggravate existing inequities in access to health services.

A global movement is emerging in response to the mental health service gap (1). International organisations are involved in this movement; the World Health Organization (WHO) launched its Mental Health Gap Action Programme (mh-GAP) in 2008 (5) and in May 2013 approved the *Global Mental Health Action Plan 2013–2020* (Sixty Sixth World Health Assembly, May 27, 2013: http://apps.who.int/gb/ebwha/pdf_files/WHA66/A66_R8-en.pdf). The response focuses on the burden of mental illness, access to mental health services, and the wellbeing of individuals living with mental illness (6).

One key strategy for addressing gaps in mental health services is building capacity at the system level (1). Capacity building is an on-going, collaborative process involving education and practical applications; it aims to foster systemic, policy, and structural change in diverse contexts. It incorporates best practices and action research, and progress depends on the strength of relationships, level of knowledge exchange, and communication between partners (7–9).

In many LMICs, little or no mental health training is available for primary healthcare (PHC) professionals. Strengthening PHC services, particularly those serving vulnerable populations, is an effective way to improve access to mental healthcare (10). Successful initiatives foster human resource capacity through comprehensive and on-going education for health and social service professionals. Several studies have demonstrated the importance of having strong, well-trained local PHC teams to better address the mental health and addiction needs of the population (11–14). Research has confirmed the success of previous mental health capacity-building initiatives in Latin America (15).

## Nicaraguan context

Nicaragua faces serious mental health needs and challenges (16–21), as shown in Table 1. The country has a population of about six million inhabitants, but less than 25% of it has access to mental healthcare. A complex political history, coupled with repeated natural disasters, has slowed progress towards modern treatment for mental health conditions, such as community-based care, instead maintaining organisational and service structures centred in psychiatric hospitals (22). Another major obstacle to progress is the lack of psychiatrists and well-trained PHC professionals in the country.

**Table 1:** Nicaraguan context: demographic and mental health facts.

Demographic facts	Mental health facts
Population: 5.9 million Poverty rate: 44.7% (2009) Literacy rate: 96.6% (2010) Infant mortality rate: 29/1,000 live births (2006) Life expectancy at birth: 74.5 years (2010)	Less than 25% of population has access to mental healthcare 1% of national health spending is allocated to mental health 90% of mental health spending is allocated to psychiatric hospitals Fewer than 3 psychiatric beds per 100,000 inhabitants 66% of municipalities have no mental health services 60% of psychiatrists are located in capital city 8% of mental health services focus on children and adolescents 2.2% of medical school class hours focus on mental health

Sources: PAHO. Health in the Americas. Washington: Panamerican Health Organization; 2012.

WHO-PAHO. Informe WHO-AIMS sobre el Sistema de Salud Mental en Nicaragua, El Salvador, Guatemala. World Health Organization and Panamerican Health Organization; 2006.

The Nicaraguan government is currently reorganising and enhancing its healthcare system. One aspect of this restructuring is the implementation of a National Health Plan (2004–2015), which identifies goals for the reform process, including: health promotion and disease prevention, increased focus on PHC, and a wider distribution of services.

The National Autonomous University of Nicaragua in León (UNAN-León) has long been promoting international collaboration as an effective approach to improving mental healthcare in Nicaragua through its Centre for Demographic and Health Research (CIDS), which has considerable experience in the fields of mental health and addictions. One of CIDS partners is the Office of Transformative Global Health at the Centre for Addiction and Mental Health (CAMH), Canada's largest mental health facility and a PAHO/WHO collaborative centre. CAMH has contributed to mental health capacity-building projects in countries throughout Latin America and the Caribbean.

Since 2008, CAMH and UNAN-León have been working together with the goal of building capacity in mental health and addictions in primary healthcare in Nicaragua. Table 2 lists the main steps of the process. Preliminary planning meetings were held at CAMH in August 2008 with CAMH staff and a leading faculty member from UNAN-León, who had expressed interest in forming a partnership. Based on the common interests and strengths of the two institutions, an agreement was made to develop a capacity-building programme for mental health in PHC in Nicaragua. This served as an excellent opportunity for mutual knowledge sharing in the context of international collaboration. In 2008, the partners finalised a ‘Letter of Understanding’ defining the institutional priorities, strategic needs and objectives for the project, particularly related to mental health and addictions in PHC. The partnership was an innovative approach to training and capacity building for interdisciplinary professionals, and laid the foundation for future action research in the region, along with possibilities for internships and exchanges for students and faculty in relevant disciplines.

**Table 2:** Timeline of the collaborative process

2008 August November	Preliminary meetings at CAMH with UNAN-León faculty member Inter-Institutional Agreement at UNAN-León with CAMH staff: ‘Letter of Understanding’
2009 March November	Leadership Training at CAMH attended by UNAN-León faculty member Participatory needs assessment First International Workshop: ‘Strengthening Mental Health in Primary Care in Nicaragua’ Participant evaluation of First Workshop
2010 March	Mental Health Symposium at CAMH with Latin American Faculty
2011 February	Second International Workshop: ‘Assessment and Treatment of Addiction in Nicaragua’ Participant evaluation of Second Workshop
2012 May	First Session of Mental Health and Addiction Diploma Program at UNAN-León
2012 November	Launch of Master Program at UNAN-León

CAMH and UNAN-León's collaborative process is based on international promising practices, specifically sustainability, negotiating, system change, local relevance, participation, and clearly-defined common objectives (23). The training was designed based on interprofessional adult education approaches (24–26). According to the Centre for Advancement in Interprofessional Education, ‘interprofessional adult education occurs when two or more professions learn with, from and about each other in order to improve collaboration and the quality of care’ (27). This approach fosters knowledge exchange, enhanced care coordination and is internationally recognised as good practice in mental healthcare (15, 26).

Some aspects of the initiative were based on previous successful partnerships between CAMH and other Latin American countries (12, 15). Over 1,000 professionals have been engaged in various trainings and collaborative projects throughout Latin America and the Caribbean, leading to improved mental health and addiction services in PHC settings, reduced stigma, and an on-going commitment to further national and international mental health capacity building (12, 15). For example, the CAMH-Chile collaboration, which started in 2003, has contributed to the improvement of the Chilean mental health system, and has supported local efforts to have Chile at the forefront of primary mental healthcare in South America. The partners view capacity building as a component of a larger transformative process of collaboration.

This article presents and analyses the results of an evaluation of the four-year collaborative process between UNAN-León and CAMH in terms of process, results, and lessons learned. Evidence of effective and respectful global health capacity building between high- and low-income country institutions is provided. The evaluation results offer a foundation/guidelines for future health-focused capacity-building initiatives based on international collaboration.

## Method

Throughout the development and assessment of this collaborative initiative, it was essential to take a modern comprehensive evaluation approach. Special consideration was also given to the programme's stage of development – which in this case is the initial stage – in order to define the most appropriate evaluation design. The *developmental approach* (28) was considered appropriate for the evaluation of this collaboration, as there is a need for on-going adaptation and model development in a context of complex nonlinear dynamics and evolving learning systems. Finally, using a mixed-methods design, partners employed a *collaborative evaluation approach* (29), as substantial participation by key stakeholders is fundamental to both implementing a collaborative initiative and evaluating it (30).

For the evaluation of key capacity-building components, the project team developed and administered evaluations and collected quantitative and qualitative data to assess the programme's efficacy in terms of fostering competencies and enhancing practices (31). Participant satisfaction, as well as attitudes, knowledge, and skills towards mental health and addictions work were key outcome measures. Based on a critical analysis of evaluation reports, key documents, and discussion among partners, the steps of the collaboration are analysed and main successes and challenges are identified.

In each step of the process, participants were selected according to profession, role in their organisation, and academic qualifications. The main targeted groups were the public health sector and academics: primary care professionals, mental health/addiction care professionals, and academics with an interest in mental health and addictions. Additionally, representatives from a number of related NGOs were invited to participate.

### Participatory needs assessment

Participants of the first training worked in groups to identify the main strengths and weaknesses regarding mental health and addictions in the Nicaraguan context in order to inform the development of the future diploma programme at UNAN-León. This process ensured that the capacity-building programme would be culturally relevant, applicable to the local context, and accessible for potential participants.

### Workshops

The data were collected from the evaluation questionnaires completed by participants at the end of the trainings. Quantitative data were collected using 5-point Likert scales, with an equal balance between positive and negative response options, thus minimising response bias. The Likert scale was chosen, as it is appropriate for survey research, and is common when measuring satisfaction, attitudes, and impressions. Qualitative data were also collected using a variety of questions. Three main areas were assessed:

- Overall satisfaction with the capacity-building programme
  - 5-point Likert scale, addressing general aspects of the workshops, with 5 representing ‘complete satisfaction’ and 1 representing ‘complete dissatisfaction’.
- Concrete learning gained from the workshops
  - 5-point Likert scale, addressing concrete learning gained from the workshops, with 5 representing ‘excellent learning’ and 1 representing ‘no learning’.
- Participants’ impressions of the workshops
  - Qualitative questionnaire, addressing questions such as ‘What did you like most’, ‘What did you like least’, and ‘What concrete learning did you gain from this workshop that will apply to your work?’, and ‘What do you recommend for future workshops?’

#### Data analysis

The quantitative data were analysed using descriptive statistics, including mean, standard deviation, minimum, and maximum. Frequencies were calculated to determine how often each response occurred. Averages were used to compile graphs to demonstrate the overall results of the data. The SPSS 15.0 statistical software package was used. Qualitative data were transcribed into a Word document. Data were checked for inconsistencies; they were then grouped according to the evaluation questions. The overall thematic analysis was based on the main evaluation questions and topics. It considered describing and clustering data, finding commonalities, and examining deviant cases. In this way, it was possible to identify key patterns and themes. Finally, a mixed-method analysis was used in such cases where data were integrated to answer the evaluation question.

#### Dissemination

During development of the programme, local stakeholders at UNAN-León were informed of the details of the process, regarding accreditation and preparation of the Diploma and Master's programmes. Recruitment and opportunities for participation in the various collaborative activities were advertised locally among relevant populations, such as the public health sector.

The collaborative team, including local and CAMH organisers developed the evaluation process. This article represents an opportunity to disseminate the results to a broader audience, focusing not only on the workshops but also on the sustainable collaborative process as a whole.

## Results

The results are presented in four sections: Participatory Needs Assessment; Workshops; Complementary Activities in Canada; and Diploma and Master's programmes.

### Participatory needs assessment

In November 2009, the partners carried out a participatory needs assessment in the context of the first workshop to inform the overall collaboration process. Forty-seven professionals contributed to the needs assessment by discussing the following topics: local strengths and weaknesses; expected outcomes regarding competencies (knowledge, skills, and attitudes); possible methodologies and recommendations for the development of the diploma programme. Table 3 summarises the main findings. Participants identified the existence of a large pool of professionals who were motivated to address mental health and addiction issues in Nicaragua and the local political support to foster mental health services in primary healthcare as some of the main strengths of their context. In terms of weaknesses, they indicated that the educational approaches being used for health professional training were not holistic and did not cover health-promotion and disease-prevention perspectives enough. Participants also identified a number of areas in which they hoped to increase their competencies during the capacity-building programme, including screening and brief interventions, psychosocial assessment and intervention techniques for individuals, families, and groups, self-care, stigma prevention, and spirituality in care, among others. For instance, one participant stated the need of having ‘self-care workshops for staff’ in health services and fostering ‘communication among all of us who work in this area’ (La necesidad de contar con ‘…talleres sobre auto cuidado de los trabajadores’ de los servicios de salud … y fortalecer la ‘…comunicación de todos los que trabajamos en esta área’). Suggestions for training methodology were provided, such as having in-class and online learning, and covering theoretical as well as clinical and research material. The results informed the content and methodology of future trainings and laid the groundwork for the future Diploma programme.

**Table 3:** Results of participatory needs assessment

Strengths	A large pool of motivated professionals working in mental health and addictions. New practical training programmes in mental health and addictions at UNAN-León. Political will to improve healthcare, using the Family and Community Health Model (MOSAFC). Strong networks within the country between primary healthcare providers and community agencies.
Weaknesses	Ineffective distribution of human and material resources. Lack of infrastructure and awareness. New training programmes do not focus enough on prevention and health promotion. Outdated psychosocial intervention approaches.
Expected Competencies	Early detection and brief intervention strategies. Psychosocial assessment and intervention techniques for individuals, families, and groups. Psychology and biology of addictions. Interdisciplinary work, including regional networking and international collaboration. Understanding of stigma, mental health promotion, and ethics/confidentiality.
Suggested Methodology for Diploma Programme	30 participants, selected via assessment test and interviewer: appropriate attitude and aptitude. Clinical as well as theoretical content, using case studies, group, and individual work. On-going process, with supplementary training each year. Health promotion focus (monitoring health trends, media campaigns, etc.). In-class and online learning, as well as research and fieldwork.

### Workshops

In 2009, the collaborative team carried out the First International Workshop, ‘Strengthening Mental Health in Primary Care in Nicaragua’, attended by 47 professionals, including nurses, social workers, psychologists, and physicians. In 2011, the team conducted the Second International Workshop, ‘Assessment and Treatment of Addiction in Nicaragua’, attended by 32 professionals, including nurses, physicians, psychiatrists, psychologists, and social workers. These workshops served to build capacity to integrate mental health and addiction care into PHC, as well as to inform the development of the Diploma programme. The results of the quantitative and qualitative components of the evaluations are summarised below.

#### Quantitative findings

*Overall satisfaction with the capacity-building programme*

**Fig. 1:**
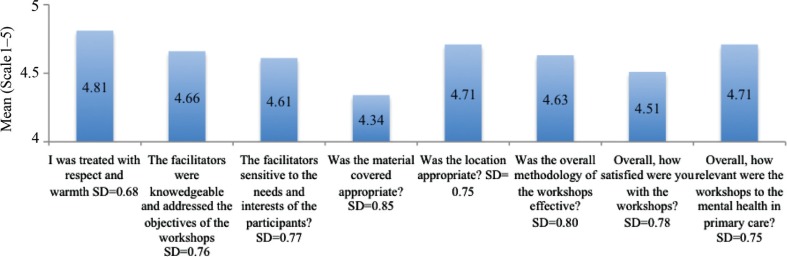
Overall satisfaction with the capacity-building programme (2009).

**Fig. 2:**
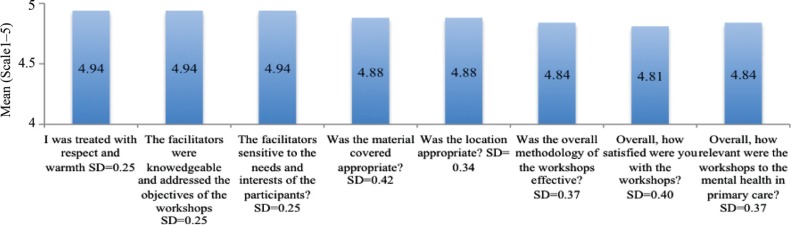
Overall satisfaction with the capacity-building programme (2011).

- 2009: The mean scores ranged from 4.3 to 4.8 for all questions, indicating overall satisfaction among participants (Fig. 1). Questions regarding respectful treatment of participants, relevance of content to work, quality of location, and competency of the facilitators were rated highest, from 4.7 to 4.8 while the quality of the materials was rated lowest, as 4.3.
- 2011: The mean scores ranged from 4.8 to 4.9 for all questions, indicating overall satisfaction among participants (Fig. 2). Questions regarding respectful treatment of participants, competency of the facilitators, and depth of content were rated highest, as 4.9, while general satisfaction was rated lowest, as 4.8.

*Concrete learning gained from the workshops*

**Fig. 3:**
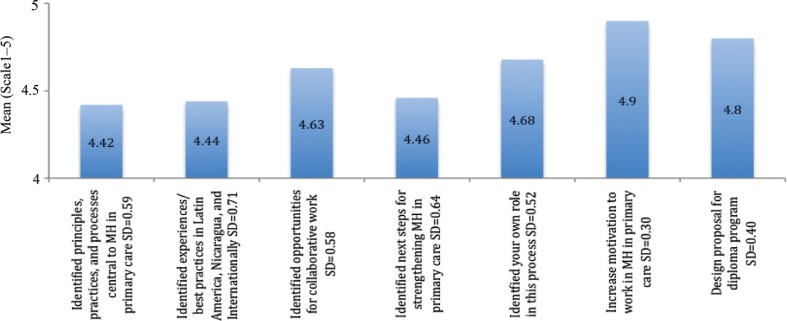
Concrete learning gained from the workshop (2009).

**Fig. 4:**
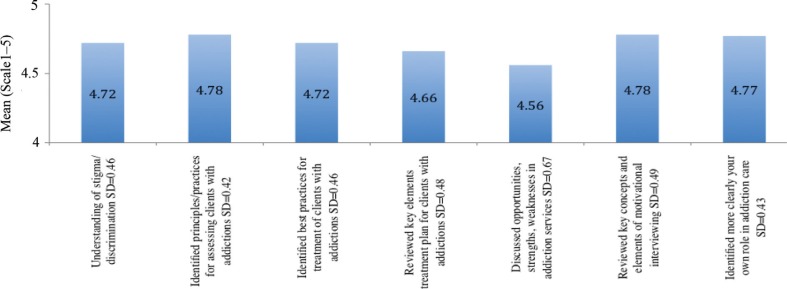
Concrete learning gained from the workshop (2011).

- 2009: The mean scores ranged from 4.4 to 4.9, indicating a high level of self-identified learning regarding the training's objectives (Fig. 3). ‘Increased motivation to contribute to mental health and addiction work in primary healthcare’ was rated highest, as 4.9, while ‘Identify examples of good practice in mental health and addiction work in primary care in Latin America’ was rated lowest, as 4.4.
- 2011: The mean scores ranged from 4.6 to 4.8, indicating a high level of self-identified learning regarding the training's objectives (Fig. 4). ‘Identified principles/practices for assessing clients with addictions’ and ‘Reviewed key concepts and elements of motivational interviewing’ was rated highest, as 4.8, while ‘Discussed opportunities, strengths, weaknesses in addiction services’ was rated lowest, as 4.6.

#### Qualitative findings

The data revealed many important themes identified by participants, with multiple occurrences on the qualitative questionnaires. The most common responses are summarised in Table 4. Some key areas of learning identified in 2009 were: inter-professional approaches to care, mental health-promotion and prevention strategies, concurrent disorders, screening and assessment, holistic care, and prevention of stigma. For instance, one participant identified as an important learning ‘team work with different disciplines’ (‘El trabajar en equipo desde las diferentes disciplinas’) and another one said that he/she learned from the ‘work experience of other people in this area’ (‘Experiencias de trabajo de otras personas sobre este tema’). The qualitative evaluation results in 2011 indicated that participants had significant learning regarding sociological basis for addiction care, assessment approaches and specific modalities to support people with substance use issues (e.g. motivational interview). The evaluation also revealed that participants felt a significant positive change in their attitudes towards people with addictions, moving towards seeing the patient as a whole person, not as an ‘addict’. A number of participants noted that they intended to try to change this attitude in their workplace or educational setting as a way to reduce stigma.

**Table 4:** Workshops: summary of qualitative findings

Year	Knowledge	Skills	Attitudes
2009	Interdisciplinary work International collaboration The national context Health promotion Disease prevention The human rights perspective	Psychosocial assessments Interprofessional work	Viewing clients as whole people Encouraging respect and empathy for clients Recognising social and economic barriers to good mental health
2011	Sociological basis for addictions care Stages of change Acceptance and recovery model Client-centred approach Treatment modalities	Motivational interviewing Narrative therapy Active listening Family integration	Viewing patients as whole people Reduced stigma regarding addiction in workplace Increased motivation

### Complementary activities in Canada

The collaboration leader from Nicaragua participated in two events in Toronto, which were organised by PAHO and CAMH.

- March 2009: *International Mental Health & Addiction Leadership Training Programme*

Aimed at improving the ability to formulate and apply innovative and viable strategies for improving mental health systems, services, and practices in various countries.

- March 2010: *Symposium on Strengthening Mental Health Plans and Services in the Americas: Scaling Up Care for Mental and Substance Use Disorders*

Aimed at improving mental health and substance use plans and services in Latin America and the Caribbean, with a particular focus on capacity building for PHC and mental health among indigenous populations.

### Diploma and Master's programmes

**Figure 5:**
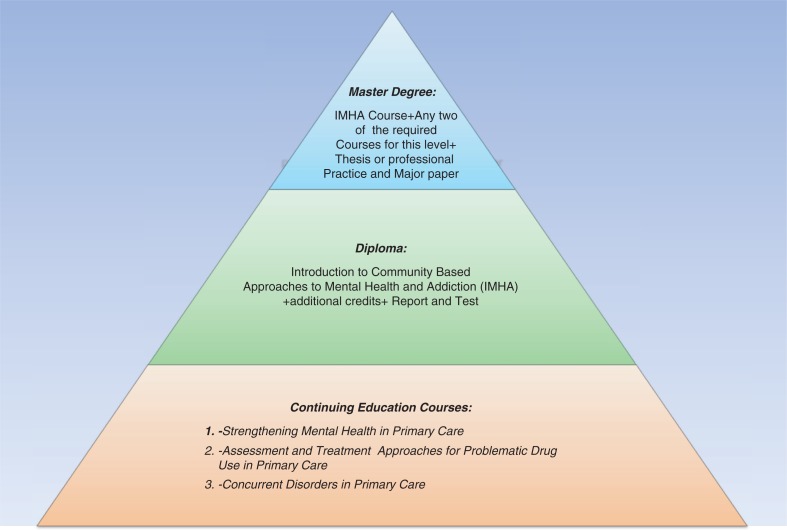
Educational pyramid for mental health and addiction in primary healthcare.

The Diploma programme is part of an overarching strategy entitled ‘An Advanced Educational Capacity Building Strategy in Mental Health and Addictions in Central America and Beyond’, which is being developed collaboratively by CAMH, UNAN-León, and the Ministry of Health of Nicaragua (MINSA). This multi-level community-based capacity-building strategy will foster interprofessional leadership and effective action to address mental health and addiction needs in PHC in the region (Fig. 5). The programme began in November 2012 and aims at fostering professional and research competencies and capacities of healthcare workers in order to more effectively address the mental health and addiction needs in primary healthcare in Nicaragua, Central America, and beyond. The programme has three key levels of training and engagement that are interrelated: (a) Continuing Education Courses; (b) Diploma; and (c) Master of Health Sciences. The initiative is directed primarily to those professionals involved in public health practice such as: physicians, dentists, nurses, psychologists, and social workers. Furthermore, to those working in planning, management, research, and evaluation in different health units and other public health agencies with main concern is related to mental health and addictions in PHC.

The strategic planning process was carried out between November 2008 and June 2011. In November 2008, CAMH and UNAN-León completed a thorough assessment and analysis of local contextual factors, identifying local strengths, needs, gaps, and opportunities for improving mental health and addictions treatment. The assessment revealed several key strengths among professionals in the region: a high level of interest in mental health; a commitment to primary healthcare; and local academic capacity and interest in international collaboration. The assessment also revealed several key areas of need: inadequate mental health services in PHC; significant challenges in suicide prevention; and insufficient mental health promotion. Based on the results of this assessment and the 2009 Participatory Needs Assessment, the organisational team developed a curriculum uniquely tailored to the demands and context of the Nicaraguan and Central American populations. The core curriculum focuses on clinical and research competencies, as well as system change regarding mental health and addictions.

The first session of the Diploma programme was held from February to September 2012, with positive results. (Data analysis is being conducted for a future article.) To ensure sustainability, the training model is designed to be easily replicated by participants, who are encouraged to train colleagues in their PHC settings. This process will further strengthen local capacity to engage in collaborative, on-going work to improve mental health and addictions services and research in PHC.

The first session of the Master's programme was launched in November 2012; it is still underway and will be evaluated upon completion in late 2013.

Additionally, the collaborative team is actively seeking out further opportunities and funding through grants and partnerships to strengthen the various components of the programme, as well as to ensure sustainability.

## Discussion

Throughout the four-year collaborative process, CAMH and UNAN-León have developed programmes that are mutually beneficial, based on knowledge exchange, sustainability, and shared respect. Together, the two centres are making long-term systemic changes that are already generating increased capacity in mental health and addictions care in PHC. Strengthening knowledge, skills, and attitudes regarding mental health and addictions will improve rates of detection and treatment, as well as reduce stigma (32, 33). Evaluating the programme thus far offers important guidelines and considerations for future capacity-building initiatives, particularly in similar contexts. The results are of interest to the international mental health and addictions community as they shed light on how to build and maintain the long-term collaborative relationship necessary for sustainable capacity building.

The conducted needs assessment process provided critical information to develop a sustainable and effective programme, which is also appropriate for Nicaragua. Some of the identified needs are similar to those addressed in other capacity-building initiatives (12, 34) and are aligned with the WHO Mental Health Gap Action Programme (mhGAP) (35). However, the high interest of participants in areas such as mental health promotion and research, as well as addiction treatment modalities, was somehow surprising. The hereby-presented capacity-building initiative addresses those areas. For instance, the focus of the 2011 workshop was on addictions. The currently implemented Diploma/Master programme covers both research and clinical practice and includes fieldwork as well as a thesis according to the particular needs and interests of each student.

Two of the learning areas that were identified by the participants are particularly important to better integrate mental health/addiction services into primary health: (i) effective interprofessional team work in healthcare; and (ii) strategies for preventing stigma towards people with mental health and/or substance use issues among health providers. Those areas are essential to enhance health systems and reach better global mental health and equity outcomes (6, 11, 27, 36).

Capacity building is an effective strategy for improving mental health and addiction treatment in PHC, encouraging sustainable change at both clinical and systemic levels. Previous research demonstrated its efficacy in a similar collaborative project between CAMH and Chilean partners (15). In the Nicaraguan context, the collaborative process between UNAN-León, CAMH, and MINSA has successfully begun to develop sustainable capacity building among health and social service professionals working in primary healthcare. The capacity-building framework employed by CAMH and Nicaraguan partners fosters a strong sense of local ownership, as it is a bottom-up rather than top-down strategy (12, 37, 38). Based on the objectives set out in the PAHO Strategy and Plan of Action on Mental Health for the Americas (39), the programme will likely serve as a model for other countries in Central America. It is also effective in terms of addressing previously identified PHC mental health challenges and training needs for Nicaragua (32, 33).

The collaborative partnership between CAMH and Nicaragua faces a number of limitations. The lack of resources committed to mental health and addiction training, service provision, and research is a significant barrier. The distance between Canada and Nicaragua also makes consistent collaborative work difficult. These challenges are not uncommon in international collaboration efforts (40, 41). Another limitation is related to contextual local will and resources: although Nicaragua has established a National Health Plan (2004–2015), the government's commitment to integrating mental health and addictions into PHC has yet to translate into extensive action. This is frequently a barrier to change in LMICs (3).

Despite these limitations, the process has achieved the objectives set out by the partners: (i) strengthening collaboration between Nicaragua and Canada to build capacity in mental health and addictions in PHC; (ii) promoting involvement of key stakeholders in the process of integration, according to existing strengths and needs; (iii) implementing the Diploma programme in mental health and addictions in PHC, with CAMH and UNAN-León faculty; and (iv) launching the sustainable, locally focused one-year Master's programme in mental health and addictions in PHC for professionals from all over Central America.

Participant evaluations of the International Workshops revealed high levels of satisfaction and evidence of concrete learning, with a strong focus on the contributions of local leaders. Participants stressed the benefits of being exposed to new perspectives like the client-centred approach, gaining technical skills for interventions, and working with an interdisciplinary team.

The collaborative team is looking forward to identifying future opportunities for capacity building and partnerships. One goal is to expand the Diploma programme into other Central American contexts. Because the model has proven to be effective in Nicaragua, it will likely also be beneficial in neighbouring countries. The design of the Diploma programme also allows alumni to deliver it to colleagues, increasing its impact. Other opportunities may include developing Master's programmes in other countries in Central America, as well as offering workshops on specific areas of mental health and addictions.

The International Workshops, the Diploma programme, and the Master's programme are all steps in the larger process of educational capacity building in Central America. By laying the groundwork for such programmes, as well as providing local professionals with the knowledge, skills, and attitudes necessary to provide effective care and to train colleagues, this process is fostering much-needed long-term systemic changes in the region.

### Lessons learned

1. Begin with a foundational comprehensive needs assessment.
2. Establish clearly identified, step-by-step, common goals between partners.
3. Involve interdisciplinary participants and faculty in the whole process to ensure relevance.
4. Develop and conduct an effective evaluation process.
5. Ensure mutual responsibility and a horizontal decision-making structure.
6. Commit to mutual respect and the principles of mental health equity.
7. Be open to local expertise and previous experience in global mental health work.

## Conclusions

To date, international collaboration between CAMH and Nicaragua has been successful. The process has led to mutual knowledge sharing, strong networking, and extensive educational opportunities. The project team is exploring opportunities for South–South partnerships, made possible by the sustainable capacity-building model that forms the foundation for the collaborative process. Gradually, CAMH will take a less active role, continuing to provide support when needed, but encouraging local partners to lead future initiatives.

Mental health is internationally recognised as a central feature of good overall health. The WHO's Millennium Development Goals set out in 2000 identified maternal health, child mortality rates, and HIV/AIDS as areas of focus for global health. All of these are closely linked with mental health, yet insufficient resources are being committed to making sustainable change in this area (42). Frichionne et al. (1) wrote, ‘the development of mental health capacity, particularly in LMICs, requires collective action based on local and global partnerships’ (p. 49). International collaboration, like that between Nicaragua and CAMH, is an essential part of fostering long-term development aimed at narrowing the mental health treatment gap. This article provides evidence of an effective and respectful global health capacity-building initiative to address a global and urgent public health issue.
